# Metzger's Challenge

**DOI:** 10.2478/v10053-008-0036-x

**Published:** 2008-07-15

**Authors:** Adam Reeves

**Affiliations:** Department of Psychology, Northeastern University, Boston MA, USA

Notice of “Laws of seeing” by Wolfgang Metzger, 1936, translated into English for the first time by Spillmann, Lehar, Stromeyer, and Wertheimer, and published by MIT Press (October, 2006).

Readers will find this an erudite and fascinating summary of the Gestalt principles known up to 1936, written to appeal to informed as well as lay audiences, and enlivened by numerous examples including some on camouflage which antedate more recent treatments. At 200-odd pages, Metzger’s book is a welcome addition to the bookshelf. The translators are especially to be congratulated on their care in translating concepts and terms as accurately as possible while still preserving the sense of excitement which permeates the work.

Gestalt Psychology was still regarded as ‘German’ by the Nazi regime in 1936, so the publication of work done by this school of Psychology was still possible at that time; however, the enormous contribution of Jewish scientists is mostly suppressed, and this should be kept in mind by the reader. Interestingly, though, it is not entirely eliminated: for example, the seminal work of Susanne Liebmann – which was excluded from standard treatments of Gestalt Psychology in English, even into the 1980s – is mentioned. Post-war editions of Metzger’s book remedied this defect.

And now, I invite you to attempt Metzger’s challenge: trying teaches one quite a bit about the operation of Gestalt factors, and one can always read the book to find answers:

“It is precisely that which naturally ‘belongs together’ that gets organized together. And what belongs together is that which ‘fits’ together, that is, that which together results in a well-organized, unitary structure. Things that by virtue of their chance position appear to ‘belong’ to something else as a ‘necessary’ component can in this way successfully disappear from our view, even though they are lying completely exposed. You can make a nice party game out of ‘openly hiding’ erasers, pencils and other things up to the size of a walking stick according to this law.”

I very much hope that the translators’ effort will inspire more translations of fundamental Gestalt texts, so that non-German-speaking readers can better appreciate this work and go beyond the simplified list of Gestalt factors which is the common text-book fare. The presentation of phenomenology and argument which allowed the Gestaltists to deduce properties of functions of the visual brain is especially important; it is in this manner that Psychology can stand on its own ground as well as provide independent and meaningful assistance to basic neuroscience.

